# Analysis of Variables that Influence the Success Rates of Induction of Labor with Misoprostol: A Retrospective Observational Study

**DOI:** 10.1055/s-0042-1744287

**Published:** 2022-04-26

**Authors:** Thayane Delazari Corrêa, Adriano Nascimento Barreto Junior, Maria Clara Mendes Batista, Mário Dias Corrêa Júnior, Henrique Vitor Leite

**Affiliations:** 1 Department of Gynecology and Obstetrics, Universidade Federal de Minas Gerais, Belo Horizonte, MG, Brazil

**Keywords:** obstetrics, misoprostol, complications of labor and delivery, induced labor, obstetrícia, misoprostol, complicações do trabalho de parto, trabalho de parto induzido

## Abstract

**Objective** Determine the predictive criteria for success in inducing labor for live fetuses using misoprostol in pregnant women. Secondarily, the objective is to determine the rates of vaginal or cesarean delivery, duration of induction, interval of administration of misoprostol, the main causes of induction of labor and indication for operative delivery.

**Methods** Medical records of 873 pregnant women admitted for cervical maturation from January 2017 to December 2018 were reviewed in a descriptive observational study of retrospective analysis, considering the following response variables: age, parity, Bishop Index, doses of misoprostol, labor induction time. Logistic regression models were used to predict success with misoprostol in non-operative deliveries.

**Results** Of the 873 patients evaluated, 72% evolved with vaginal delivery, 23% of the cases were cesarean, 5% forceps or vacuum-extractor. For non-operative delivery the predictive variables at admission were age, parity, gestational age and dilation. During hospitalization, fewer vaginal touches, amniotomy or amniorrhexis with clear fluid lead to a shorter induction time and a greater chance of non-operative delivery. False positives and false negatives of the model were always below 50% and correct answers above 65%.

**Conclusion** At admission, age less than 24 years, previous normal births, lower the gestational age and greater the dilation, were predictive of greater probability of non-operative delivery. During hospitalization, the less vaginal touches and occurrence of amniotomy/amniorrhexis with clear liquid indicate shorter induction time. Future studies with a prospective design and analysis of other factors are necessary to assess the replicability, generalization of these findings.

## Resumo

**Objetivo** Determinar os critérios preditivos para o sucesso na indução do trabalho de parto para fetos vivos utilizando misoprostol em gestantes. Em segundo lugar, o objetivo é determinar as taxas de parto vaginal ou cesáreo, duração da indução, intervalo de administração de misoprostol, as principais causas de indução do trabalho de parto e indicação para parto operatório.

**Métodos** Foram revisados os prontuários de 873 gestantes internadas para amadurecimento cervical entre janeiro de 2017 e dezembro de 2018 em um estudo descritivo observacional de análise retrospectiva, considerando as variáveis-resposta: idade, paridade, Índice de Bishop, doses de misoprostol, tempo de indução do trabalho de parto. Modelos de regressão logística foram utilizados para prever o sucesso com misoprostol em partos não operatórios.

**Resultados** Dos 873 pacientes avaliados, 72% evoluíram com parto vaginal, 23% dos casos foram cesáreos, 5% fórceps ou vácuo-extrator. Para o parto não operatório as variáveis preditivas na internação foram idade, paridade, idade gestacional e dilatação. Durante a internação, um menor número de toques vaginais, amniotomia ou amniorrexe com líquido claro, levam a menor tempo de indução e maior chance de parto não operatório. Falsos positivos e falsos negativos do modelo sempre foram inferiores a 50% e respostas corretas acima de 65%.

**Conclusão** Na internação, idade menor que 24 anos, ocorrência de partos normais anteriores, menor idade gestacional e maior dilatação, foram preditivos de maior probabilidade de parto não-operatório. Durante a internação, o menor número de toques vaginais, amniotomia/amniorrexe com líquido claro indicam menor tempo de indução. Estudos futuros com design prospectivo e análise de outros fatores são necessários para avaliar a replicabilidade, generalização desses achados.

## Introduction

Labor induction is one of the most performed obstetric interventions and refers to techniques of stimulation of the uterine contractions that will lead to labor. 1 According to the World Health Organization (WHO), in an assessment of maternal perinatal health, 9.6% of births worldwide need to be induced. 2

The decision to induce labor is made when the continuity of pregnancy is associated with increased maternal or fetal risk, and there is no contraindication to vaginal delivery. 1 Successful induction of labor depends on the maturity of the cervix, which is generally assessed using the Bishop index, the best predictor of success for vaginal birth nowadays. 3 Several techniques for cervical ripening and labor induction are evaluated with the aim of reducing the cesarean section rates, with the available mechanical and pharmacological options. 4

The main mechanical methods are: artificial rupture of membranes (amniotomy), membrane sweeping, and cervical dilators (laminaria and Krause method). 5 Pharmacological methods include prostaglandins (PGE2: dinoprostone or PGE1: misoprostol), selective modulators of progesterone receptors, oxytocin, and nitric oxide (NO) donating compounds. 6 7

Misoprostol, a synthetic analogue of prostaglandin E1, has been used for labor induction since the 1990s and has a plasma half-life of less than one hour when administered vaginally. 8 9 It has been shown to be an effective stimulator of the myometrium of the gravid uterus by several studies. The use of misoprostol for induction of labor is still *off label* . This drug was initially approved by the FDA in an oral form (Cytotecs, Pfizer) to reduce the risk of ulcers induced by non-steroidal anti-inflammatory drugs (NSAIDs). However, this medication has been used for the past 30 years in the third trimester of pregnancy for cervical ripening and labor induction, being orally, vaginally, rectally, and sublingually applied, in high or low dose regimens, although the ideal route of use is still unknown. The doses initially used for induction of labor were empirical and ranged from 25 µg every 3 to 6 hours, to 200 µg in a single dose intravaginally or orally. 10

Thus, as labor induction rates increase, it is of clinical importance to clearly determine the different variables that influence the safety and effectiveness of methods for inducing labor in pregnant women. As the appropriate doses of misoprostol for preparation and induction of labor in pregnant women with live fetuses are not well established, in the proposed study, the primary objective was to determine the predictive criteria for the success of labor induction with the use of misoprostol in pregnant women at the Otto Cirne Maternity Hospital of the Hospital das Clínicas of UFMG. Our secondary objective was to determine the rates of vaginal or cesarean delivery, mean duration of induction, interval of misoprostol administration, the main causes of induction of labor, and indications for operative delivery.

## Methods

A descriptive observational study of retrospective analysis was performed, with a review of the clinical records of pregnant women admitted for labor induction at the Otto Cirne Maternity of the Hospital das Clínicas of UFMG from January 2017 to December 2018.

Data were collected through the analysis of electronic and physical records of patients admitted for delivery. Pregnant women candidates for cervical ripening underwent maternal and fetal evaluation, confirming the absence of contraindications to induction of labor and vaginal delivery. The gestational age of all patients was determined by date of last menstruation or earlier ultrasound, anamnesis, and clinical examination.

The inclusion criteria in this study were patients followed-up at our service, with medical and obstetric indications for the labor induction with misoprostol in live fetuses. Exclusion criteria were based on our primary design, which was to study misoprostol labor induction in women with live fetuses. Therefore, in addition to the exclusion of women hospitalized for induction with a dead fetus on admission, induction with oxytocin without the use of misoprostol and women who had the induction initiated by legal interruption were also excluded, since fetal vitality and the newborn's outcome were not important and limiting factors of conduct for the obstetrician in these situations. Furthermore, women with contraindications to induction of labor or vaginal delivery were also excluded, as established in the HC-UFMG Obstetrics Protocol: history of previous uterine rupture, history of gynecological surgery on the uterine body (such as intramural myomectomy), active genital herpes, total placenta previa or vasa previa, cord prolapse, anomalous fetal presentations (except in fetal descent), macrosomia with estimated fetal weight greater than 4 kg, invasive cervical cancer, patient's refusal, non-reassuring fetal pattern, anomalous pelvis, some fetal congenital anomalies such as neural tube and/or abdominal wall closure defects with good neonatal prognosis, and fetal tumors that determine fetal-pelvic disproportion. Women who had used oxytocin after misoprostol and whose fetuses died during hospitalization were not excluded from this study.

Data collected from medical records were: age, parity, gestational age at admission and at delivery, days of hospitalization, maternal morbidity, induction indication, uterine height on admission, Bishop index on admission, total number of misoprostol tablets used, total number of doses of misoprostol used, analgesia, obesity according to the body-mass index (BMI), use of oxytocin during induction, maximum dose of oxytocin, type of delivery, time of delivery after beginning of induction, indication of operative delivery, Apgar index at birth (first minute) and Apgar after 5 minutes, fetal condition at birth (alive or dead), newborn weight, amniorrhexis, and clear fluid appearance, considered in admission and during labor. Maternal morbidity and indication for induction were described according to the medical records but for analysis purposes, groups were defined as described below.

Regarding maternal morbidity, as pregnant women admitted to the Hospital das Clínicas are primarily considered “High Risk” (∼60%), around 85 different comorbidities were identified and divided into 8 groups: no comorbidities, hypertensive disorders, diabetes, mental disorders, heart disease, kidney disorders, infectious diseases, and “other”: cholestasis, asthma, obesity, myasthenia gravis, thrombophilia, hypothyroidism, hearing loss, anemia, rheumatic fever, factor XI deficiency, congenital deafness, dermatopolymyositis, autoimmune thrombocytopenia, coagulopathy, Crohn disease, rheumatoid arthritis, adrenal adenoma, Cushing syndrome, isoimmunization, neoplasm, previous history of thrombosis, drug use, liver transplantation, systemic lupus erythematosus (SLE), epilepsy, myomatosis, pheochromocytoma, cholelithiasis, need for cerclage in pregnancy, alcoholism, Graves ophthalmopathy, hyperthyroidism, idiopathic thrombocytopenic purpura (ITP), antiphospholipid antibody syndrome (APLS), pituitary adenoma, sickle cell trait, smoking during pregnancy, maternal hydrocephalus, Hodgkin lymphoma, Von Willerbrand disease, ascites, vitiligo, pulmonary hypertension, isthmus-cervical incompetence, Turner syndrome, and history of bariatric surgery.

One point to be raised would be the lack of consensus on the concept of successful induction of labor. For example, for successful induction, the National Institute for Health and Clinical Excellence (NICE) considers achieving a vaginal delivery within 24 hours. 11 However, the WHO considers the rate of cesarean sections as an indicator of success. 2 And the Society of Obstetricians and Gynecologists of Canada (SOGC) considers vaginal delivery between 24 and 48 hours of induction as a success. 9 Other authors add “uncomplicated vaginal delivery,” or “reaching the active phase of labor.” 3 In our study, labor induction was considered successful when non-operative vaginal delivery occurred, without the use of forceps or vacuum extractor. Procedures with absence of uterine contractions, changes in the cervix, or complications during labor culminating in a cesarean section were considered as unsuccessful labor inductions.

The hospital chosen for the research was the Otto Cirne Maternity of UFMG's Hospital das Clínicas (UFMG-HC). This is a general, public, university hospital, which is a reference center in highly complex care for the Unified Health System of Minas Gerais (UHS/MG), with a monthly average of 190 births at the time of this study. The UFMG-HC protocol for misoprostol use determines a maximum dose of 275 mcg divided into 8 doses, as follows: 1 tablet of 25 mcg via the vaginal route every 4 hours in the first 5 doses, and 2 tablets of 25 mcg via vaginal (50 mcg) every 6 hours on the sixth, seventh, and eighth doses. Therefore, data was collected considering the number of doses, ranging from 1 to 8 doses; but the total number of pills inserted can range from 1 to 11 pills. In our study, the dose of misoprostol used was in accordance with the American College of Obstetricians and Gynecologists (ACOG) guidelines, which recommends a misoprostol dosage of 25 mcg every 3 to 6 hours (50 mcg every 6 hours may be appropriate in some situations). 5

This research project was approved by the UFMG Research Ethics Committee (CAAE 06358919.7.0000.5149–Number 3.278.259, April 23rd, 2019) and does not present any conflict of interests.

As for the descriptive analysis, data from all patients were initially collected and recorded in Excel (Microsoft CO. Redmond, WA, USA). The statistical software R (R Foundation for Statistical Computing, Vienna, Austria) was used to set up a database and perform the statistical analyses. Data related to categorical variables were analyzed in frequency tables, which have both absolute and relative frequency. For the numerical variables, the measures of central tendency used were the mean and median; as measures of variation, we used the standard deviation (SD), the minimum value, the maximum value, and in some cases the limits of the 95% confidence intervals (95% CI) of the average.

The analyzes of the quantitative variables with the response variable (non-operative delivery) were performed using box plots 12 and the non-parametric statistical Mann-Whitney test, 13 also known as the unpaired Wilcoxon test, using the wilcox.test function in R. As for the qualitative variables, the analyzes were performed using bar graphs and the Yates chi-square test, with the chisq.test function in R. 14

In a second step, multivariate logistic regression analysis was performed, aiming to create a predictive statistical model. Multivariate (or multiple) analyzes were performed using logistic regression techniques, with the glm function in R, which generates a final equation that can be used for future predictions, in addition to saying how much each variable influences (increases or decreases) the probability of occurrence of the event of the response variable. 14 We used the backward variable selection method, also known as variable elimination. 13 The final significance level chosen was 0.01 and not 0.05, due to how this selection method causes a possible underestimation bias of *p* -values. It is noteworthy that regardless of the result of the bivariate analyses, all variables entered the first model. Finally, the quality of the predictions of the models was evaluated through sensitivity, specificity, percentage of correct answers, and false positives and negatives.

## Results

**Fig. 1:**
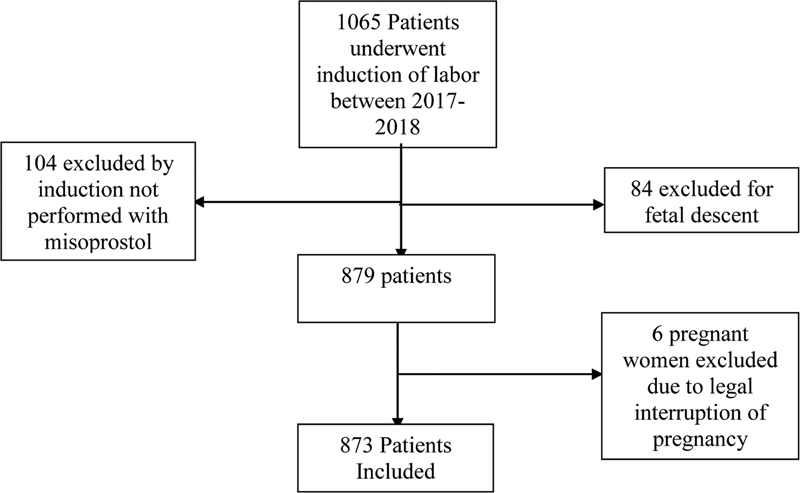
Flowchart of patients selected for the study.

We selected 1065 patients hospitalized for labor induction at the Hospital das Clínicas of UFMG from January 2017 to December 2018. Among those, 84 pregnant women hospitalized with fetal failure and 104 pregnant women whose induction was not performed with the aid of misoprostol were excluded. The final analyzed sample contains 873 patients. No patient refused to undergo the induction process after medical advice and clarification of doubts ( Fig. 1 ).

Table 1 shows the mean age of patients, Bishop score on admission, number of misoprostol doses used in total, time of delivery after induction onset, Apgar score at birth and gestacional age at delivery.

**Table 1:** Characterization of patients regarding mean age, Bishop score on admission, total number of misoprostol doses, time of delivery after induction onset, Apgar score at birth, and gestational age at delivery

	Mean	95% CI of the mean	Minimum – maximum
Age (years)	27.66 ± 6.83 (27)	(27.21; 28.12)	13–48
Bishop index at admission	1.59 ± 1.32 (1)	(1.50; 1.68)	1–6
Total number of misoprostol doses	3.59 ± 2.26 (3)	(3.44; 3.74)	1–10
Delivery time after beginning of induction (hours)	21.22 ± 13.30 (18.25)	(20.33; 22.10)	1–109
Apgar index at birth	7.85 ± 1.88 (8)	(7.72; 7.97)	0–10
**Gestational age at delivery**		**N (%)**	
Birth < 37 weeks		99 (11.4)	
Birth 37–41 weeks and 6 days		773 (88.5)	
Birth ≥ 42 weeks)		1 (0.1)	
Total		873 (100)	

**Abbreviations:** 95% CI, 95% confidence interval.

The body mass index (BMI) of the participants was also evaluated; however, for only 139 of the participants was this information present in the medical record. Thus, we obtained a sample mean equal to 31.8, sample standard deviation equal to 6.69 and median equal to 31. Regarding the number of previous vaginal deliveries, 53.15% of the pregnant women had no previous vaginal delivery, approximately 25% of the patients had one previous vaginal birth, 12.03% had two previous vaginal deliveries, and almost 5% had 3 or more previous deliveries. Table 2 characterizes the patients regarding maternal morbidity, with 59.22% of the pregnant women having some comorbidity.

**Table 2:** Maternal morbidity

		Frequency
Groups	n	%
No comorbidities	356	40.8%
Hypertensive disorders	317	36.3%
Others	205	23.5%
Diabetes	127	14.6%
Infectious diseases	59	6.8%
Mental disorders	21	2.4%
Kidney disorders	21	2.4%
Heart diseases	20	2.3%
Total participants	873*	–

**Note:** *Patients could have more than one comorbidity, so the sum of the number of pregnant women in each group is greater than 873, which is the total number of participants.

Table 3 shows the delivery outcome. It is clear that most patients who participated in the survey had non-operative vaginal delivery, which corresponds to just over 72%. The second most common type of delivery was the cesarean operation with almost 23% of the cases, followed by the forceps vaginal delivery, with approximately 4% of the deliveries, and finally the extraction vacuum vaginal delivery, which had just under 1% of the cases.

**Table 3:** Childbirth outcome

Frequency		
Type of delivery	n	%
Cesarean delivery	199	22.8%
Vaginal delivery	632	72.4%
Vaginal delivery by forceps	34	3.9%
Vaginal delivery by vacuum extractor	8	0.9%
Total	873	100%

The time of delivery after the beginning of induction was also evaluated, which was timed from the insertion of the first misoprostol tablet until the birth of the newborn. We observed that 67.81% of the patients delivered within 24 hours and 26.92% delivered within 12 hours of the beginning of induction as an outcome. A considerable amount also presented delivery from 24 to 36 hours after the beginning of induction, approximately 18%. Only 4% gave birth 48 hour after the start of induction. The total average time of delivery after beginning of induction was 21.22 hours, with a sample deviation equal to 13.3. The smallest value identified was equal to 1 and the largest value equal to 109 hours. With the 95% confidence interval of the mean, we found a lower limit of 20.33 and an upper limit of 22.10. With the analysis of Table 4 , we can see the main indications for labor induction, the main reason being hypertensive disorders, which affected approximately 37.46% of the patients. Antepartum amniorrhexis was the second most recurrent reason, found in 23.02% of the participants. Gestational age (18.33%) was the third most recurrent reason for labor induction, and comorbidities related to diabetes also had a significant frequency, in approximately 12.6% of the patients. The other indications occurred in a maximum of 10% of the patients. As each patient could have more than one reason for induction, the sum of frequencies in Table 5 is not equal to the total number of participants.

**Table 4:** Indication of labor induction

	Frequency	
Indication	n	%
Hypertensive disorders	327	37.5%
Gestational age ≥ 41 weeks	160	18.3%
Antepartum amniorrhexis	201	23%
Diabetes	110	12.6%
Fetal indication	87	10%
Fetal malformations	57	6.5%
Others	55	6.3%
Severe maternal comorbidity	32	3.7%
Infectious diseases	29	3.3%
Total of participants	873	–

**Notes:** Fetal malformations: fetal heart disease, trisomy 13, trisomy 18, cystic adenomatoid malformation of the lung (MAC) type II, Dandy-Walker syndrome. Fetal indication: fetal macrosomia, intrauterine growth restriction (IUGR), fetal flow centralization, fetus large for gestational age (LGA), oligohydramnios, pelvic presentation, polyhydramnios.

**Table 5:** Indication of surgical delivery

		Frequency	
Indication	n	%	Total %
Acute fetal distress	84	34.9%	9,6%
Induction failure	46	19.1%	5.3%
Maternal exhaustion	24	10%	2.8%
Cephalo-pelvic disproportion	24	10%	2.8%
Secondary arrest of dilation	21	8.7%	2.4%
Cesarean on request	8	3.3%	0.9%
Placental abruption	8	3.3%	0.9%
Macrosomy	5	2.1%	0.6%
Not informed	4	1.7%	0.5%
Acute fetal distress and maternal exhaustion	3	1.2%	0.3%
Pelvic presentation	2	0.8%	0.2%
Maternal heart disease	2	0.8%	0.2%
Twin pregnancy	2	0.8%	0.2%
IUGR with altered doppler	1	0.4%	0.1%
Induction failure and fetal macrosomia	1	0.4%	0.1%
HELLP syndrome	1	0.4%	0.1%
Provenance of hands	1	0.4%	0.1%
Fetal risk of intrapartum vaginal death	1	0.4%	0.1%
Acute fetal distress and breech presentation	1	0.4%	0.1%
Acute fetal distress and induction failure	1	0.4%	0.1%
Acute fetal distress and secondary arrest of dilation	1	0.4%	0.1%
Total	241	100%	27.6%

**Abbreviations:** IUGR, intrauterine growth restriction; HELLP, hemolysis, elevated liver enzymes, and low platelets. **Notes:** Induction failure: absence of labor after insertion of 8 doses of misoprostol or 11 tablets. Acute fetal distress: described in medical record as “persistent fetal bradycardia,” “non reassuring fetal state “, “late deceleration in CTG” and “prolonged deceleration in CTG.” The number of operative vaginal deliveries is 42 (4,8%); antepartum c-sections, 50 (5,7%); intrapartum c-sections, 149 (17%).

Table 5 refers to the indication for operative delivery (cesarean section or instrumentalized vaginal delivery, with the use of forceps or vacuum extractor). The most recurrent indications are related to the group of acute fetal distress (34.85%), already presented, as well as induction failure (19.09%), cephalopelvic disproportion (CPD, 9.96%), and maternal exhaustion (9.96%).

The logistic regression analysis considering the outcome “nonoperative delivery” was performed. To adjust the final model, the database was first divided into a training base and a test base, the first having 70% of all observations (611) and the second 30% (262). Then, the selection of variables was made using the training base and following the backward method, in which the variables are extracted one by one. The equation for the probability of non-operative delivery is presented as supplementary material . The percentage of success of the predictive model in both the test base and the training base was 79.1%. This gave us confidence that there was no overfitting of the data, and the final result considered the entire database. However, although the original database contains 873 patients, as the records of some patients had missing values for some variables, 856 patients remained for evaluation of the model's prediction, as shown in Table 6 .

**Table 6:** Logistic regression forecast results for non-operative childbirth: final template for admission and hospitalization variables

Type of delivery	Template (Expected Response)		
(Response observed)	Operative delivery	Non-operative delivery	Total
Operative delivery	84	149	233
Non-operative delivery	30	593	623
Total	114	742	856

**Notes:** sensitivity = 95.2%; specificity = 36.1%; hit percentage = 79.1%; false positives = 20.1%; false negatives = 26.3%.

In another template for nonoperative delivery, we chose to consider only the variables available at the time of patient's admission. The equation for the probability of non-operative delivery is presented as supplementary material . It is important to note that the three variables in common in the two results above (age, previous normal births, and gestational age at admission) have almost identical coefficients in the two models, indicating the robustness of the results. The percentage of accuracy of the predictive model was 73% in the test base and 76% in the training base, also not indicating a very big difference that could indicate an overfitting of the model to the data. Thus, the final results considered the entire database. Taking into consideration that the records of some patients had missing values for some variables, 871 patients remained for evaluation of the template's prediction, as shown in Table 7 .

**Table 7:** Logistic regression prediction results for non-operative childbirth: final template for admission variables only

Type of delivery	Template (expected response)		
(Response observed)	Operative delivery	Non-operative delivery	Total
Operative delivery	56	185	241
Non-operative delivery	33	597	630
Total	89	782	871

**Notes:** sensitivity = 94.8%; specificity = 23.2%; hit percentage = 75.7%; false positives = 23.7%; false negatives = 37.1%.

## Discussion

The aim of this study was to determine the predictive criteria for success in labor induction with the use of misoprostol, in addition to determining the rates of vaginal birth or cesarean operation, mean duration of induction, interval of misoprostol administration, the main causes of labor induction, and indication of operative delivery.

The association between cesarean operation and induction is reinforced by daily obstetric practice, and it is a common belief that induction of labor increases the risk of cesarean operation. However, using the appropriate comparison group, studies show that induction of labor is actually associated with a small decrease in this risk. 15 The labor induction rate between the years 2017 and 2018 at Hospital das Clínicas of UFMG was 27.8%, and the cesarean rate was 37.87% in the total number of deliveries performed. 16 Of the induced deliveries, we had a rate of caesarean section of 22.79% found in the study, which is significantly lower than the total group of patients monitored in our hospital.

This finding is in line with what is registered in the literature, and in a meta-analysis the cesarean rate was quite variable between the compared trials, with an overall trend of reduction with vaginal misoprostol (34 trials, RR (Relative Risk) 0.95, 95% CI (Confidence Interval) 0, 87 to 1.03). 17

Regarding the time of delivery after the start of induction, we observed that almost 70% of the patients delivered within 24 hours, and approximately 27% delivered within 12 hours of the start of induction.

A randomized clinical trial demonstrated a higher proportion of women who delivered within 12 hours and within 24 hours using misoprostol combined with mechanical dilation using a Foley tube. 18

The American College of Obstetricians and Gynecologists (ACOG) suggests that the appropriate dosage of misoprostol is 25 mcg every 3 to 6 hours (or 50 mcg every 6 hours, in some situations), the SOGC recommends 50 mcg orally with a glass of water or 25 mcg vaginally every 4 hours, while WHO recommends 25 mcg of oral misoprostol every 2 hours or 25 mcg of misoprostol vaginally every 6 hours for labor induction. 9 In our study, the dose of misoprostol used is in accordance with the ACOG, and proved to be adequate with satisfactory results.

A review on labor induction showed that the success of induction with vaginal birth increases with gestational age. 3 In our study, the most frequent types of pregnancies in the sample are those considered early term and full term, with 37.57% and 33.33% of the situations respectively, which may have influenced the best outcome.

In a prospective observational study, the main cause of induction was pregnancy ≥ 41 weeks, 29.8%, 17.9% with antepartum amniorrhexis, elective induction in 9.5% of the cases, followed by preeclampsia in 8.5% of cases, 8.1% with oligohydramnios, severe maternal morbidity in 7.7%, diabetes in 3.8%, severe fetal morbidity in 3.3%, and other causes < 2%. 19 In our study, however, the main reason for induction were the hypertensive disorders, which affected approximately 37.46% of the patients. Followed by antepartum amniorrhexis in 23.02% of the participants. Gestational age was the third most recurrent reason for labor induction, in 18.33% of the patients, and comorbidities related to diabetes also had a significant frequency, in approximately 12.6% of the patients. The other indications occurred in 10% of the patients at most. This difference in relation to the literature may have occurred because the Hospital das Clínicas of UFMG is a high-risk referral unit.

As for the indication of cesarean, in a study of labor induction with oxytocin, misoprostol, or both, it was found that acute fetal distress played an important role in the indication of cesarean, with 35.1% correlation rates, followed by CPD with 23.4%, and 16% of induction failure. 20 In the present study, we found very similar results, with the most recurrent indications related to the group of acute fetal distress (34.85%), with induction failure in second place (19.09%), followed by CPD (9.96%), and maternal exhaustion (9.96%).

It should be noted that, in our study, these indications are not only for cesarean section, but also instrumentalized vaginal delivery, in which case the main cause was maternal exhaustion.

For induction to be successful, we generally take into account the maturity of the cervix, which is assessed using the Bishop index, the best predictor of success for vaginal birth nowadays. 3 A review that considered more than 40 articles correlated the Bishop index at the beginning of induction with its outcome, concluding that it would be a poor predictor and should not be used to decide whether or not to induce labor. 21 At the moment, however, this index remains the main tool for evaluating the uterine cervix at the beginning of induction. Our model proposes to complement this index, as it includes other variables that were not considered as predictors until now.

Regarding the logistic regression models found, for non-operative delivery, the model showed that at the time of admission, the younger maternal age, more previous normal deliveries, lower gestational age, and greater dilatation, all contribute for a higher probability of this patient undergoing non-operative delivery, which confirms the results in the literature. During hospitalization, the lower number of vaginal touches, in addition to the occurrence of amniotomy, amniorrhexis (on admission or hospitalization), and appearance of clear fluid, were related to a higher occurrence of non-operative delivery.

For the models where the answer is non-operative delivery, the percentage of correct answers was 79% (admission and hospitalization variables) and 76% (only admission variables), which are considered high values. The percentages of false positives (35% and 42%) and false negatives (21% and 21%) were less than 50% in both models. Sensitivity was excellent in both models (95%), but specificity was low in both with 36% considering all variables (admission and hospitalization) and 23% using only admission variables. One of the reasons for this difference between sensitivity and specificity is the fact that the models predicted more non-operative deliveries than the actual total. Thus, it can be said that there was a “difficulty” of the models in identifying and predicting operative deliveries.

Overall, both logistic regression models designed here had difficulty predicting the least frequent outcome, which was operative deliveries (241 deliveries out of 873). On the other hand, false positives and false negatives were always less than 50%, and the percentage of correct answers was greater than 65%, indicating that the predictions made by such models are always more likely to be right than wrong.

A strength of this study would be that the overall clinical volume of the studied hospital and cesarean rates did not change significantly over the years spanning the study period, making the confounding factor related to temporal trends less likely. Another relevant point of this study was that we arrived at final models for predicting childbirth, with both admission and hospitalization variables.

As this is a retrospective study with review of medical records, some of the necessary patient data were not present in the medical records. Another important limitation of this study is that, although we had statistical power to detect differences in time from induction to delivery, for most outcomes—including cesarean operation, and adverse maternal and neonatal outcomes—we did not have the statistical power to discern potentially important differences between groups.

## Conclusion

At admission, factors such as younger maternal age (age < 24 years), more previous normal births, lower gestational age, and greater dilatation, were all associated with a higher probability of undergoing non-operative delivery. During hospitalization, fewer vaginal touches, amniotomy and amniorrhexis with clear fluid, and shorter labor induction time were associated with a greater chance of non-operative delivery. However, despite the percentage of false positives and false negatives being always below 50% and that of correct answers being above 65%, the final models had difficulty predicting the outcome “operative delivery” because it was less frequent.

Furthermore, in our study, labor induction with misoprostol had a 15% lower cesarean incidence compared with the overall cesarean rate of our hospital in the study's period, with most patients (almost 70%) giving birth in up to 24 hours after initiation of induction, using up to 4 doses of the tablet.

The most recurrent indications for operative delivery and the main causes of labor induction in this study were similar to those found in the literature, the second differing only in the frequency and order of the results found, a fact that may have occurred because the Hospital das Clínicas of UFMG is a high-risk reference unit. Future studies in different environments, with a prospective design and analysis of other factors are needed to assess replicability, generalization of these findings, and improved prediction rates.
