# Supplementary material for: Analysis of Variables that Influence the Success Rates of Induction of Labor with Misoprostol: A Retrospective Observational Study
Source: Rev Bras Ginecol Obstet. 2022 Apr 26;44(4):327–35. doi: 10.1055/s-0042-1744287 (PMC9948114; doi:10.1055/s-0042-1744287)
Supplement: Supplementary file 1 — Supplementary Material [file 10-1055-s-0042-1744287-s210342.pdf]

Supplementary material

1. Equation for the probability P(Y = 1) of non-operative delivery:

$$P(Y=1)=\frac{e^{6{,}945-0{,}093x_1+0{,}631x_2-0{,}027x_3-0{,}166x_4+3{,}735x_5+3{,}943x_6+1{,}016x_7}}{1+e^{6{,}945-0{,}093x_1+0{,}631x_2-0{,}027x_3-0{,}166x_4+3{,}735x_5+3{,}943x_6+1{,}016x_7}}$$

The response variable is $Y = 1$ (non-operative delivery) and $Y = 0$ (operative delivery), and the predictor variables were $x1$ = age, $x2$ = previous vaginal deliveries, $x3$ = gestational age at admission (days), $x4$ = number of vaginal touches, $x5$ = amniotomy, $x6$ = amniorrhexis, and $x7$ = clear fluid appearance.

2. Equation for the probability P(Y = 1) of non-operative delivery:

$$P(Y=1)=\frac{e^{9{,}242-0{,}082x_1+0{,}653x_2-0{,}026x_3+0{,}383x_4}}{1+e^{9{,}242-0{,}082x_1+0{,}653x_2-0{,}026x_3+0{,}383x_4}}$$

The response variable is $Y = 1$ (non-operative delivery) and $Y = 0$ (operative delivery), and the predictor variables were $x1$ = age, $x2$ = previous vaginal deliveries, $x3$ = gestational age at admission (days), $x4$ = cervical dilation on admission.

 © 2022. Federação Brasileira de Ginecologia e Obstetrícia. All rights reserved.
